# Predicting Innovative Work Behaviour in an Interactive Mechanism

**DOI:** 10.3390/bs12020029

**Published:** 2022-01-28

**Authors:** Samina Afrin, Tarik Raihan, Ahmed Ishmum Uddin, Md. Aftab Uddin

**Affiliations:** 1 Department of Human Resource Management, University of Chittagong, Chattogram 4331, Bangladesh; samina@cu.ac.bd (S.A.); ishmum.hrm@std.cu.ac.bd (A.I.U.); 2 School of Management, Universiti Sains Malaysia, Gelugor 11800, Malaysia; 3 Department of Management, University of Chittagong, Chattogram 4331, Bangladesh

**Keywords:** creative self-identity, creative self-efficacy, creative process engagement, innovative work behavior, creative climate, ICT

## Abstract

The purpose of this paper is to investigate the impacts of employees’ creative self-efficacy (CSE) and creative self-identity (CSI) on their innovative work behaviour (IWB), with the indirect effects of creative process engagement (CPE) and creative climate (CC). Following the deductive reasoning approach, the study was conducted on IT-based firms in Bangladesh. A total of 348 surveys were collected using a multi-item questionnaire. The collected data were then analyzed using structural equation modeling (SEM). The study reveals a significant relationship between CSE and IWB, and CSI and IWB. It further explores the significant mediating effects of CPE and the moderating effects of CC on CSE and IWB, and CSI and IB, relationships. Based on the premise of interactionist perspectives on creativity, this study contributes to the literature proposing a distinctive model comprising five variables to investigate employees’ IWB from a multi-level perspective. This integrated model, using predictors from multiple levels, supports the theoretical assumption that IB results from employees’ CSE, CSI, and, finally, CPE. Distinct from the other literature, the study also portrays the moderating and mediating impact of CC and CPE simultaneously.

## 1. Introduction

There is no other option in this competitive world but to ‘innovate or die’; if not, organizations will lose their position to competitors [1,2,3]. To reap the best possibilities in a volatile environment, the creative process engagement (CPE) of the workforce, together with the divergent process of creativity capabilities, have become a thriving force in innovative work behaviour (IWB) [4,5]. Although there is a clear disparity between the concepts of CPE and IWB, the two notions are often delineated identically. Studies have asserted that it is impossible to achieve IWB without the engagement of creative mindsets in the creative process [6,7,8]. Recent studies have posited the importance of creative self-efficacy (CSE) and creative self-identity (CSI) as crucial factors in stimulating employees’ CPE toward their IWB [1,9]. Furthermore, studies also suggested that the presence of creative climate (CC) matters to significantly bridge a person’s belief in their creative ability with CPE and IWB [8,10]. Therefore, integrating the theoretical insights of the self-efficacy theory, the self-identity theory, and the interactionist perspective of creativity, the degree of CPE toward IWB in an organization depends on the inclination of employees to be engaged in the creative process with a high level of CSE and CSI, which can be significantly moderated by the CC [1].

Consequently, researchers have endeavored to incorporate complementary variables such as CSI, CC, CSE, CPE, and IWB [11]. Individuals with high self-efficacy and self-identity are perceived to be more committed to their actions [12]. Moreover, within a creative climate, a strong determination works in the back of the minds of the employees to undertake more creative practices, with an intention to practice more thought-provoking activities [8]. Henceforth, in the headway of creativity and innovation domains, the contributory role of CPE in the nourishment of IWB has been recognized and validated by various research; whereas, very few studies have been conducted to demonstrate the psychosomatic aptitude of employees, nor the intervening factors that are the preconditions of wielding IWB [13,14,15]. In this regard, this study aims to investigate both the direct effect of CSE and CSI on IWB, and the mediating effect of CPE on IB. The noteworthy contribution of this study is embodied in the creative engagement of employees in recognizing and resolving problems with constructive solutions, which necessitates a higher degree of CSE and CSI that can be significantly moderated and mediated by the creative climate and engagement in the creative process, respectively.

The study enriches the CPE and IWB literature by adding several valuable aspects with novel discernment [11,16,17]. First, the study aims to develop an inclusive model to rationalize the relationship between the different variables of CSE, CSI, CC, and CPE, as well as their successive impacts on IWB. Particularly, the moderated mechanism with the inclusion of moderator (CC) and mediator (CPE) using both CSE and CSI as the direct predictor of CPE leading to IWB is newly investigated, which provides notable insight for the conceptualization of the CPE-IWB relationship. Second, the study explores the state of CPE and IB in the eastern context, whereas most previous research has been conducted in the western context [18,19,20]. Finally, it sheds light on the IWB of employees from the perspective of small and medium enterprises (SMEs), with the holistic representation of diverse industries such as IT, production and services, and light engineering, which significantly phase out the generalizability problems of previous findings [21,22].

## 2. Hypothesis Development

### 2.1. Relationship between Creative Self-Efficacy and Innovative Behaviour

Self-efficacy refers to the intrinsic confidence of individuals in achieving particular goals that shape both the quality and amount of the efforts they make towards successful attainment of specific tasks [23,24]. CSE has long been identified as an influential contributory aspect to IWB in both the individual and organizational contexts, and individuals with high self-belief on their creative capability eventually make things happen [25,26]. In a sense, IWB can be defined as a product of creative thinking ability, which brings out something new and unique. The study by Qiang et al. [27] asserts that IWB requires extensive thinking ability in creative ways to achieve sustainable outcomes and is the result of individuals’ belief in their ability to make things happen [28]. Therefore, the following hypothesis was formulated:

**Hypothesis** **1** **(H1).** *There is a significant relationship between creative self-efficacy and innovative behaviour.*

### 2.2. Relationship between Creative Self-Identity and Innovative Behaviour

CSI is another dominant factor contributing to IWB, which refers to the self-perceived concept of creative identity [29]. In this research, CSI is the societally ascribed perceived image that, along with the perceived self-image, influences individuals to behave in line with the role identities accredited to them and portrays the distinctiveness and separation from others (who we think ourselves to be others) [30,31,32,33]. Creative self-identity enhances an individual’s endeavors in IWB, which instinctively are the result of the perceived images shaped by how people assess themselves and how others assess them [34,35,36]. The path of individual interaction, peer support, and engagement in IWB is directed by role identity [37,38]. Therefore, the following hypothesis is proposed:

**Hypothesis** **2** **(H2).** *There is a significant relationship between creative self-identity and innovative behaviour.*

### 2.3. Relationship between Creative Process Engagement and Innovative Work Behaviour

Most studies in the field of creativity have focused on uncovering the IWB of organizations, rather than investigating the factors responsible for creativity and innovation [2,39]. Contemporary research in the field of creativity now emphasizes the process of engaging in creative endeavor, which ultimately leads to the generation of creative outcomes [17,22,40]. CPE has been identified as the rational process of involvement in creative endeavor through the identification of underlying problems; searching for and encrypting relevant data consistent with the problem in question; and, finally, generating an alternative solution for the problem [22,41]. The aspirations of employees to engage in IWB necessitate significant and active involvement in identifying and exploring problems and constructing alternative solutions [17,42,43]. Moreover, organizational success in a competitive atmosphere hinges on the voluntary involvement of its employees through vigorous engagement in framing creative ideas and devising constructive roadmaps to gain a competitive edge [44]. Therefore, based on the previous literature, it can be stated that if employees dedicate themselves to CPE, they are more likely to develop IWB [13,17,45]. Consequently, the following hypothesis is proposed:

**Hypothesis** **3** **(H3).** *Creative process engagement has a significant influence on employees’ innovative work behaviour.*

### 2.4. Mediating Role of Creative Process Engagement

Employees with CSE tend to be more inclined towards achieving dynamic results than those with low CSE [46]. Being involved in various creative processes, individuals with CSE tend to bring about changes through psychological, behavioural, and subjective investment of their time and attention by identifying problems; probing, collecting and encoding information; generating feasible solutions; and, finally, implementing them [42,47]. Devloo [48] reported that employees’ CSE contributes to IWB because they perceive the situation to be under their control. It has been observed in different settings that if self-belief is high, then engagement in complex creative processes for innovation will follow [18]. Based on the theoretical discussion above, the following hypothesis is posited.

**Hypothesis** **4** **(H4).** *Creative process engagement mediates the relationship between creative self-efficacy and innovative behaviour.*

CSI enables employees to be confident about their abilities to be creative in their work and encourages them to engage in innovation in order to reaffirm the identity consigned to them [49]. Possession of CSI influences engagement in the creative process, through which IWB is ultimately facilitated. As hypothesized in role identity theory, an individual socially identified as being creative is perceived to have a duty to be engaged in the creative process, thus delivering IWB [9,30]. As IWB is inevitable for organizational growth, employees’ engagement in the creative process is essential [50]. Likewise, in the absence of a creative outlook, it is evident that IWB is implausible [1,51]. Based on the above literature, the following hypothesis is suggested.

**Hypothesis** **5** **(H5).** *Creative process engagement mediates the creative self-identity-innovative behaviour relationship.*

### 2.5. Moderating Effect of Creative Climate

Adaptation to volatility and sustainable competitiveness requires a favourable working climate [8]. Creation and the execution of innovative ideas are possible only when the organization can ensure a creative atmosphere [52]. The underlying concept of CC is grounded in IPC theory, and its impact has been assessed through the lens of previous studies [53,54]. Employees with CC feel a psychological compulsion to undertake risk and devise novel ideas, feeling comfortable to construct and propose innovative solutions to problems [55,56]. On the contrary, an absence of CC dilutes the process of developing creative aptitude in employees and subsequently weakens their commitment to CPE [4,43]. Previous studies have found a significant impact of CC on the relationship between CSE and CPE [4,57].

The underlying notion of CSI is that the expectations of an employee to gain personal and social advantage in an institutional setting encourage them to engage in the creative process [58]. Therefore, individuals with CSI will be more conscious of their role and strive to exhibit creative slants to redefining problems with creative solutions [51]. Notably, CC facilitates psychosomatic safety, allowing employees to develop more creative ideas; establish risk-taking aptitudes; and encourage curiosity to explore uncovered areas [59]. On the contrary, a climate hostile to creativity has an adverse effect on the creative involvement of employees and adversely affects the CSI-CPE relationship because of demoralization [4,43]. Hence, if an organization can warrant CC, employees with CSI will be able to display more eccentric endeavors with risk-taking attitudes and yield more constructive outcome [52,60]. Earlier studies have also demonstrated a significant influence of CC on the CSI-CPE relationship [17,59]. Therefore, the following hypotheses are proposed:

**Hypothesis** **6** **(H6).** *Creative climate moderates the influence of creative self-efficacy on creative process engagement.*

**Hypothesis** **7** **(H7).** *Creative climate moderates the influence of creative personal identity on creative process engagement.*

## 3. Research Methods

### 3.1. Research Design

The research employed a quantitative research method with a deductive reasoning approach [61,62]. The perceptual value of the constructs was measured using a multi-item scale adopted from previous studies. In order to obtain valid feedback, the original questionnaire in English was translated into the native language (Bangla), and then translated back into English under the guidance of an expert panel through the back-translation process [63]. Until the affirmation of original representativeness of the statements, the process was repeated [64].

### 3.2. Data Collection Procedure and Sample Characteristics

The study used the convenience sampling method to collect cross-sectional data because the convenience sampling is the viable solution when the population is homogenous in nature and questionnaires need to reach respondents quickly [65]. The data were collected from ICT and Internet service provider firms listed in the Bangladesh Association of Software and Information Services (BASIS). After receiving approval from the concerned authority (BASIS), 600 questionnaires were distributed among the firms through personal visits and e-mail. Furthermore, the researchers attempted to eliminate response bias by considering each organization as single unit, and accordingly delivered two sets of instruments, one for top-level management to rate their subordinates, and another for employees to rate the organizational climate.

Three hundred and forty-eight matched replies from both managers and employees were received after sending repeat emails and making personal visits, a response rate of 58%, which has been found to be acceptable in similar contexts [1,8,18]. Due to erroneous responses, missing data, outlier problems and unmatched cases, 9 responses were discarded, leaving 339 for the final analysis. Table 1 shows that the majority of the respondents were men (258, 76%), with only 81 (24%) women. Out of the 339 respondents, the highest number (179, 53%) had obtained a postgraduate degree, followed by 116 (34%) with a bachelor’s degree, and the remainder (44, 13%) with other degrees. Remarkably, 184 (54%) of the respondents were in the 25–35 year old age group, with 82 (24%) in the 35–45 year old group, 50 (15%) in the 18–25 group, and the remainder (23, 7%) in the above 45 age group. Finally, in terms of experience it was found that most of the employees (145, 43%) had had a job tenure of between 5–10 years, followed by 83 (24%) with above 1 year, 81 (24%) with above 10 years, with the remainder (30, 9%) having above 15 years job experience.

### 3.3. Response Bias

The study observed that the application of cross-sectional data curtailed the relationship strength of the structural connotation and inhibited the generalizability of the research outcomes [66]. The authors gave assurances to the respondents that their identity would be kept anonymous and their responses recorded in a confidential manner, which ultimately encouraged them to provide their responses accurately and prevented the problem of social desirability biasness [66,67]. Additinally, we ran the Harman [68] one factor test, with the results showing that first factor explained only 27.14%, or less than 50%, of the total variance (76.93%). To identify and explore the association between variables that surpassed 0.90, we scrutinized the correlation matrix by following Bagozzi’s method; it was found that 0.641 was the highest association between any two variables [69,70].

Moreover, alternative model analysis was conducted to establish model-fit measures (Table 2). A comparison between the various factor models showed that the 5-factor model (x^2^/df = 1.278; GFI = 0.927; CFI = 0.987; TLI = 0.986; SRMR = 0.031; RMSEA = 0.029) generated a better fit index than the 1-factor model (x^2^/df = 8.969; GFI = 0.501; CFI = 0.63; TLI = 0.593; SRMR = 0.13; RMSEA = 0.154) [71]. Finally, the existence of a collinearity problem was tested by estimating the variance inflation factors, none of which exceeded the threshold limit (10.00) [65,72]. Therefore, on the basis of the evidence, it can be asserted that there were no issues related to method or response bias.

### 3.4. Measurement Tools

All the measurement instruments were adopted from previous studies. Each item was measured on a 5-point Likert-type scale ranging from 1 (strongly disagree) to 5 (strongly agree). The survey measure of Jaiswal and Dhar [10] was used to estimate CSE. The CSI of employees was measured by using the tool developed by Karwowski [73]. The scale for creative climate was adopted from the work of Kim and Yoon [74], and finally, the measurement tools of CPE and IWB were adopted from the work of Zhang and Bartol [22] and Zhang and Begley [75], respectively.

### 3.5. Analytical Tools

The study aimed to analyze the collected data using different concurrent statistical tools such as IBM SPSS 23, IBM SPSS AMOS 23, and Microsoft Excel. Structural equation modeling (SEM) was used to justify the robustness of the outcomes by incorporating both the measurement model and structural model. Moreover, the study also included confirmatory factor analysis (CFA) and structural path estimates [76,77].

## 4. Results

### 4.1. Measurement Issues

**Figure 1:**
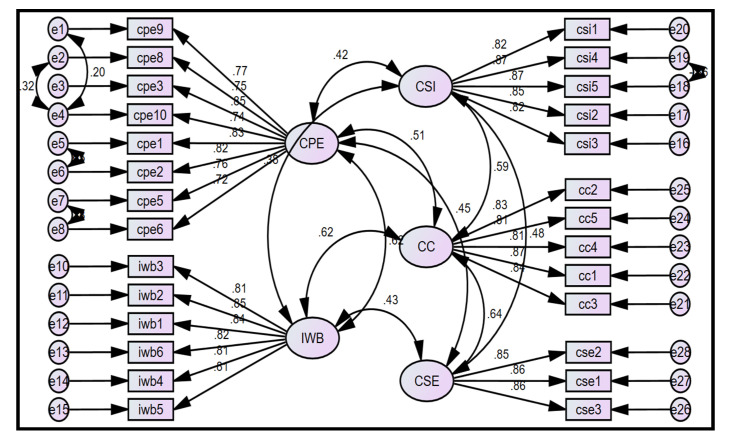
Confirmatory factor analysis.

In order to substantiate the measurement model, the item fitness suitability was assessed by examining the CFA along with the reliability and validity estimates. Figure 1 shows that the average regression weight of each construct was more than 0.70, which is above the threshold limit. Moreover, CFA also prescribes that the study generates a better fit index (x^2^/df = 1.278; GFI = 0.927; CFI = 0.987; TLI = 0.986; SRMR = 0.031; RMSEA = 0.029).

The estimation of reliability and validity can be seen in Table 3, which shows that the lowest values of composite reliability and average variance extracted (AVE) are 0.893 (>0.80) and 0.611 (>0.50) respectively, which are above the minimum threshold limits [62,77]. Additionally, the estimates also show that the square root of the AVE of all the latent variables is higher than the correlation value of a variable with other variables, which demonstrates that there is no problem of discriminant validity [78,79]. Therefore, it can be confirmed that there is no concern over measurement issues [76,77].

### 4.2. Hypothesis Testing

#### 4.2.1. Direct Effects

**Figure 2:**
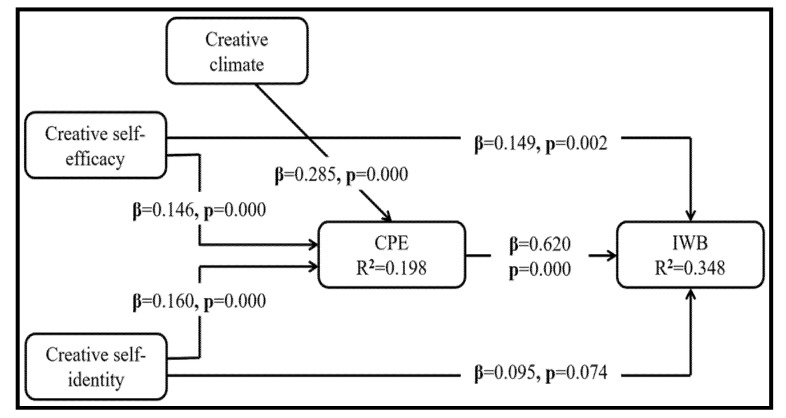
Structural model. CPE = Creative process engagement; IWB = Innovative work behaviour.

With reference to Hypotheses 1 and 2, the direct influence of CSE and CSI on IWB was estimated. In Figure 2, the assessment shows that a positive influence of CSE on IWB and that the relationship is significant (β = 0.149, *p* = 0.002). Therefore, Hypothesis 1 is supported. In Hypothesis 2, it is proposed that there is a positive influence of CSI on IWB. Surprisingly, in contrast to the hypothesis, the estimation showed an insignificant relationship (β = 0.095, *p* = 0.074). Therefore, Hypothesis 2 is not supported. Moreover, in Hypothesis 3, it is proposed that CPE has a positive influence on IWB. In line with the hypothesis, it was found that there was a significant relationship between CPE and IWB (β = 0.620, *p* = 0.000). Therefore, the hypothesis is supported.

#### 4.2.2. Mediation Effect

The bootstrap method in PROCESS macros was run to examine the mediating effect of CPE on the influence of CSE and CSI on IWB. According to the criteria, the bias-corrected confidence interval must not contain zero for the specific indirect effect to have a mediation effect [80]. Table 4 shows the mediation effects. H4, regarding the mediation effect of CPE on the association between CSE and IWB, is supported because the confidence interval does not include zero. Since the direct effect of CSE on IWB still remains significant, there is a partial mediation. Likewise, in H5, the influence of CPE on the association between CSI and IWB is also supported. There is full mediation, since the direct effect of CSI on IWB is insignificant in the structural model.

#### 4.2.3. Moderating Effect

Table 5 shows the moderating effect of CC on the influence of CSE and CSI. Model 1 indicates the effect of the control variables on CPE, with none found to be significant. Models 2 and 3 demonstrate the direct effects of CSE, CSI, and CC on CPE, which were found to be significant (*p* < 0.05). Moreover, we investigated the moderating effect of CC on the CSE-CPE (Hypothesis 6) and CSI-CPE (Hypothesis 7) relationships. The results shown in Table 5 (model 6) indicate that Hypothesis 6 (the moderating effect on CSE-CPE) and Hypothesis 7 are not supported.

**Figure 3:**
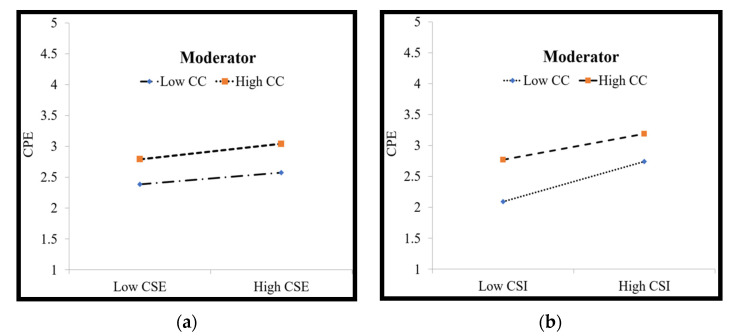
(**a**) Moderation effect of CPE on CSE-CPE; (**b**) Moderation effect of CPE on CSI-CPE.

Furthermore, we plotted the estimates in Figure 3a,b, which confirmed that a higher level of CC does not make a significant difference to the influence of CSE and CSI on CPE.

## 5. Discussion

The prevailing evidence that reflects the existence of a significant relationship between CSE and IWB [81] is clearly demonstrated in our findings in relation to Hypothesis 1 (β = 0.149, *p* = 0.002). Hypothesis 2 proposes that CSI positively predicts IWB. Surprisingly, in contrast to the hypothesis and previous research findings, the estimate does not support the significant prediction (β = 0.095, *p* = 0.074) [8,38]. Mediating variables such as CPE may have a stronger impact on predicting the relationship indirectly than the direct relationship between CSI and IWB [18]. In Hypothesis 3, we investigate the influence of CPE on IWB. The estimated results show that the effect is significant (β = 0.620, *p* = 0.000) and consistent with the findings of previous studies [1,18].

This study explored an additional area by investigating the mediating effect of CPE on CSE and IWB, and on CSI and IWB. According to our results, CPE mediates the relationship between CSE and IWB, strengthening the notion that employees who are self-efficacious in terms of creativity tend to engage themselves in the creative process, which ultimately facilitates IWB [82]. Therefore, the dependence of IWB on individuals’ CSE through CPE is both theoretically and statistically supported. Most studies [8,42,60] argue that employees who identify themselves as creative contribute significantly towards innovation [83].

Furthermore, the mediating effect of CPE on the relationship between CSI and IWB is also statistically significant, which implies that self-identified creative individuals portray innovative behaviour by engaging themselves in the creative process [84]. Moreover, the full mediation effect of CPE on the influence of CSI on IWB indicates that the influence of CSI influences IWB via CPE more than the influence of CSI on IWB alone. This means that individuals with a preoccupied mindset of being capable flourish their identity to achieve innovation through involvement in the creative outcome yielding process.

The study also sheds light on the moderating effect of CC on the relationships between CSE and CPE, and CSI and CPE. The results demonstrate that CC does not moderate the CSE-CPE relationship, thus indicating that CC is possibly not relevant to the study context. On the other hand, CC was also not found to significantly moderate the relationship between CSI and CPE. According to the results, individuals with creative identity do not associate themselves with the creative process more or less, given a favorable creative climate [53]. The findings postulate that the existence and persistence of creative individuals over ensuring a creative climate is more crucial for employees to engage in creative pursuits [8].

### 5.1. Theoretical Contributions

In the context of the 21st century, organizations need to invest a substantial proportion of their resources in the development of their employees, as well as fostering innovation through research in order to overcome the challenges of competition and to adjust to the knowledge- driven society. The outcomes of the study augment the existing pool of literature by adding several insightful contributions. First, in the field of creativity and innovation literature, most studies have striven to explore the independent role of CSE and CSI on CPE and IWB, but very few have explored the moderated and mediated impact of CC and CPE on the concerned relationship. Holistically, the study has endeavored to connect the integrative impact of CSE and CSI on IWB through the moderation and mediation mechanisms in the hypothesized relationship. Second, the role of creative individuals with efficacy and self-identity in creative behaviour in the south Asian context has not been portrayed adequately in the literature. This study, therefore, enhances the prevailing knowledge through its significant contribution to the creative literature. Third, the study has endeavored to demonstrate the application of the interactionist perspective from the employee viewpoint, with its multi-faceted implications in measuring employees’ IWB.

### 5.2. Managerial Implications

Achieving organizational sustainability in today’s competitive atmosphere clearly necessitates the effective management of employees’ creativity, which eventually facilitates the innovative outcome of organizations. Most ICT firms are facing innovation challenges from their competitors due to the frequent changes in consumer preferences. Employees can play an important driving role in taking advantage of opportunities from market dynamics through their resilience and innovativeness [85]. Managers can apply the finding of the research from different perspectives. First, the outcomes of the study show a direct positive relationship between CSE and CSI, and IWB. Therefore, managers can stimulate the creative aptitude of employees in different ways in order to improve their CPE towards IWB. Second, the study has explored the mediating role of CPE in the CSE-IWB and CSI-IWB relationships. If managers can provide enough flexibility, autonomy, and opportunities for career progression, employees will show enthusiasm for engaging in creative endeavor, entailing higher self-efficacy and self-identity, which will ultimately lead to IWB. Third, in light of the findings of the research on insignificant moderating influence of CC on exogenous and endogenous variables, the mangers or administrators need to remodule their conventional supervision styles [8]. The policy guidelines of managers and policy-makers need diversion from over-emphasizing CC to the acquisition of talents with heightened CSE and CSI. Moreover, top level management can strive to redesign their recruitment and selection policies by paying more attention to the creative aptitude of candidates during the selection procedure in order to synergizing their creative performance. Moreover, the study also sheds lights on the justification of the need to develop creative individuals in the ICT industry. Finally, the results signify that if an organization can facilitate a pro-creative workplace environment, CPE and CSI will make a greater contribution to CPE and IWB. Therefore, fostering IWB in IT professionals needs organizational nurturing in order to create a proactive and creative organizational climate.

### 5.3. Limitations and Future Research Directions

Though the use of both reports’ survey and other precautions during the data collection process prevents the estimated results from being affected by common method bias, the study also has some underlying limitations. First, the data were collected only from ICT firms listed in BASIS, which weakens the general applicability of the findings. Future researchers could minimize this problem by incorporating diverse organizations from varying industries in the data collection process. Second, in order to examine the causal inferences, the study used cross-sectional data to draw inferences, which may have generated erroneous causal relationships. Future studies could overcome this limitation by using experimental or longitudinal research paradigms [35]. Third, the research model was developed by adopting constructs from different previous studies, which were conducted mostly in western settings. This research examined the implications of the model and attempted to suggest empirical support in the South Asian context. Further studies could demonstrate the comparative scenario between the eastern and western contexts. Fifth, the study projected IWB and CPE by using CSE and CSI as independent predictors. Future studies could incorporate more relevant factors such as intelligence, openness, conscientiousness, team efficacy, personality, and leadership, which could be responsible for envisaging the creative aptitude of employees.

## 6. Conclusions

Using the lens of multiple theories, the present study aimed to examine the predictors of IWB in a moderated mechanism with both rated samples from IT-related firms in an emerging country, Bangladesh. It also tested the mediating role of CPE in the CSE-IWE and CSI-IWB relationships in the light of multi-theory perspectives. The existing body of literature in the field of creativity will be strengthened by this research through its fascinating findings. The study reveals that CSE and CSI have unswerving impact on IWB. Moreover, it demonstrates the full mediating role of CPE on the CSE-IWB and CSI-IWB affiliations, while also revealing the insignificant moderating impact of CC on the CSE-CPE and CSI-CPE relationships. Subsequently, sustainable IWB is strengthened when CPE plays a dominant mediating role in the CSE-IWB and CSI-IWB relationships.

## Figures and Tables

**Table 1:** Demographic information (n = 339).

Variable	Classifications	Frequency	Percentage
Gender	Male	258	76
	Female	81	24
Education	Graduate	116	34
	Postgraduate	179	53
	Other	44	13
Age	Above 18	50	15
	Above 25	184	54
	Above 35	82	24
	Above 45	23	7
Experience	Above 1 year	83	24
	Above 5 years	145	43
	Above 10 years	81	24
	Above 18 years	30	9

**Table 2:** Alternative model evaluation.

Alternative Model	CMIN/DF	GFI	CFI	TLI	SRMR	RMSEA
5-Factor model (CSI, CSE, CC, CPE, IWB)	1.278	0.927	0.987	0.986	0.031	0.029
4-Factor model (CSI + CSE, CC, CPE, IWB)	2.953	0.817	0.911	0.900	0.084	0.076
3-Factor model (CSI + CSE + CC, CPE, IWB)	4.417	0.724	0.843	0.826	0.074	0.101
2-Factor model (CSI + CSE + CC, CPE + IWB)	6.356	0.614	0.752	0.727	0.103	0.126
1-Factor model (CSI + CSE + CC + CPE + IWB)	8.969	0.501	0.63	0.593	0.13	0.154
Threshold	1.00–3.00	>0.90	>0.95	>0.95	<0.08	<0.06

CPE = Creative process engagement; IWB = Innovative work behaviour; CSI = Creative self-identity; CC = Creative climate; CSE = Creative self-efficacy, GFI = Goodness of Fit; CFI = Comparative fit index; TLI = Tucker Lewis index; SRMR = Standardized root mean square residual; RMSEA = Root mean square error of approximation.

**Table 3:** Reliability and validity estimates.

Latent Variable	CR	AVE	CPE	IWB	CSI	CC	CSE
CPE	0.926	0.611	0.782				
IWB	0.927	0.679	0.615 ***	0.824			
CSI	0.927	0.716	0.425 ***	0.375 ***	0.846		
CC	0.919	0.695	0.513 ***	0.615 ***	0.593 ***	0.834	
CSE	0.893	0.735	0.449 ***	0.429 ***	0.482 ***	0.641 ***	0.857

*** *p* < 0.001, CPE = Creative process engagement; IWB = Innovative work behaviour; CSI = Creative self-identity; CC = Creative climate; CSE = Creative self-efficacy; CR = Composite reliability; AVE = Average variance extracted.

**Table 4:** Mediation effect.

Hypothesis	Path	Estimate	Standard Error	*p*-Value	CI	
					LL	UL
H4	CSE → CPE → IWB	0.206	0.040	0.000	0.138	0.291
H5	CSI → CPE → IWB	0.220	0.039	0.000	0.150	0.300

CPE = Creative process engagement; IWB = Innovative work behaviour; CSI = Creative self-identity; CSE = Creative self-efficacy; CI = Confidence interval; LL = Lower limit; UL = Upper limit.

**Table 5:** Moderation effect.

Variable	Model 1	Model 2	Model 3	Model 4	Model 5	Model 6
(Constant)	1.880	1.288	0.988	0.910	0.978	0.857
Age	0.028	0.023	0.029	0.014	0.015	0.011
Tenure	0.064	0.046	0.048	0.046	0.045	0.046
Education	0.019	−0.010	−0.017	−0.012	−0.012	−0.017
Gender	−0.143	−0.124	−0.108	−0.096	−0.094	−0.091
Creative self-efficacy		0.345 ***	0.242 ***	0.142 **	0.110	0.058
Creative self-identity			0.248 ***	0.150 **	0.149 **	0.267 **
Creative climate				0.252 ***	0.218	0.282 **
CSE X CC					0.015	0.043
CSI X CC						−0.058
R^2^	0.026	0.182	0.239	0.282	0.282	0.284
∆R^2^		0.156	0.057	0.043	0.000	0.002

*** *p* < 0.001 ** *p* < 0.05; CSI = Creative self-identity; CC = Creative climate; CSE = Creative self-efficacy.

## Data Availability

Not applicable.
